# Minor hysteresis patterns with a rounded/sharpened reversing behavior in ferromagnetic multilayer

**DOI:** 10.1038/s41598-018-22810-y

**Published:** 2018-03-13

**Authors:** Duy-Truong Quach, Duc-Thang Pham, Duc-The Ngo, The-Long Phan, Seung-Young Park, Sang-Hyuk Lee, Dong-Hyun Kim

**Affiliations:** 1 Faculty of Basic Sciences, University of Transport and Communications, Hanoi, 10000 Vietnam; 2 Faculty of Engineering Physics and Nanotechnology, VNU University of Engineering and Technology, Hanoi, 10000 Vietnam; 3 Department of Physics, Chungbuk National University, Cheongju, 28644 South Korea; 4 School of Materials, University of Manchester, Manchester, M13 9PL United Kingdom; 5 Department of Physics and Oxide Research Center, Hankuk University of Foreign Studies, Yongin, 17035 South Korea; 6 Spin Engineering Physics Team, Korea Basic Science Institute, Daejeon, 34133 South Korea

## Abstract

Hysteresis of ferromagnetic system exhibits a fundamental stimulus-response behavior, thereby casting all the important macromagnetic system parameters such as coercivity, nucleation field, saturation magnetization, and hysteresis loss. Recently, increasing attention has been paid to exploration of relatively less understood minor loop behavior, since faster operation of magnetic devices is inevitably accompanied by minor hysteresis behavior from cycling among unsaturated ferromagnetic states. Here, we report our microscopic investigation of unusual minor hysteresis loop behavior, represented by rounded or sharpened response of minor hysteresis loop of (CoFeB/Pd)_4_ multilayer film. It is observed that rounded and sharpened response in the minor hysteresis response could be manifested under proper conditions. The minor loop behavior has been systematically investigated by direct microscopic magnetic domain observation using magneto-optical Kerr microscopy. The rounded response of magnetization at the reversing external field along the minor hysteresis curve, so far neglected or considered as one of ‘unusual’ behaviors, has been found to be elaborately controllable by tuning the reversing field strength and the field sweep rate for multilayers with low repeat numbers. Variable roundedness of the minor hysteresis loop is understandable based on the analysis of magnetic domain dynamics such as domain nucleation and the domain wall velocity.

## Introduction

Ferroic materials exhibit a hysteresis loop^1^. Particularly in ferromagnetic materials, hysteresis plays a key role in understanding the macroscopic nature of the ferromagnetic system, where a hysteresis response depends strongly on a magnetic field history^2^. When a ferromagnetic system is swept by an external field with the maximum field strength enough to saturate the system, the hysteresis becomes a major hysteresis loop. Major hysteresis is known to be macroscopically reproducible since the loop represents macroscopic magnetic states starting from and ending up with afull magnetization saturation^3^. In case of major hysteresis responses, it has been well known that the loop shape of the major hysteresis mostly depends on the global feature of the ferromagnetic systems such as saturation magnetization, magnetic anisotropy, and exchange stiffness of the materials^2^. For instance, the major hysteresis loop is known to be controllably shifted by adjusting the exchange bias^4^.

On the other hand, a minor hysteresis loop is observed when a ferromagnetic system is under a cycling field with the maximum field not enough to saturate the system. Since minor hysteresis loops are believed to include rich context of magnetic response properties of the system, numerous studies have been devoted to understand various minor loop behaviors^5–11^. In contrast to the major hysteresis loop, minor loops exhibit several interesting characteristics. Since the ferromagnetic system is driven from a partially saturated state, the loop becomes significantly sensitive to field profiles. For instance, unlike major loops, minor loops tend to be not always reproducible due to different microscopic magnetic configurations at an unsaturated state even with the same macromagnetic parameters, which provides intriguing unusual properties such as a cumulative loop growth^7–10^. These unique features of the minor hysteresis response will be essential for further in-depth application of ferromagnetic material. Especially, in case of nanostructured ferromagnetic materials, it has been reported that microscopic response under external fields such as domain structure and relaxation behavior could sensitively depend on the detailed ferromagnetic nanostructured composition^12^. Therefore, understanding a rather complex nature of minor loop behaviors still remains technological as well as scientific challenges, while a useful analysis technique such as the first-order reversal curve has been recently applied to fingerprint the minor loop responses of various ferromagnetic systems^13–15^.

However, the microscopic investigation of minor loop behavior still lacks in providing a general aspect for a full understanding. It has been rather known that ‘usual’ minor loops show a sharp corner at reversing field where magnetization increases under an increasing field^16–18^. Very interestingly, an ‘unusual’ minor hysteresis loop behavior has been recently reported, where the magnetization decreases under an increasing field along the minor hysteresis loop, leading to a rounded response of magnetization around the reversing field point^5^. The unusual behavior of the rounded minor hysteresis response at the reversing field was roughly ascribed to a continued domain wall expansion by thermal activation, while further details with a quantitative analysis are yet unexplored. It should be mentioned that the rounded minor hysteresis response has seemingly been experimentally observed previously^5,7,11^, however, without much attention being paid since the effect was quite modest and marginal.

In this work, we systematically investigate the rounded and sharpened minor hysteresis response with directly monitoring microscopic domain patterns for (CoFeB/Pd)_4_ and (Co/Pt)_5_ multilayers, claiming that, based on the quantitative analysis of direct domain observations, the ‘unusual’ rounded response behavior is substantially manifested and not unusual in fact, but could be generally observed in most of ferromagnetic systems when specific conditions are matched. Also, it has been found that the rounded response could be systematically engineered and manifested by controlling the external field profiles such as reversing field strength and field sweep rate. Lastly, a novel ‘unusual’ sharp response of the minor loop around the nucleation corner has been observed to exist, adding a richer characteristic of the minor loop response.

## Results

### Unusual minor hysteresis loops

**Figure 1:**
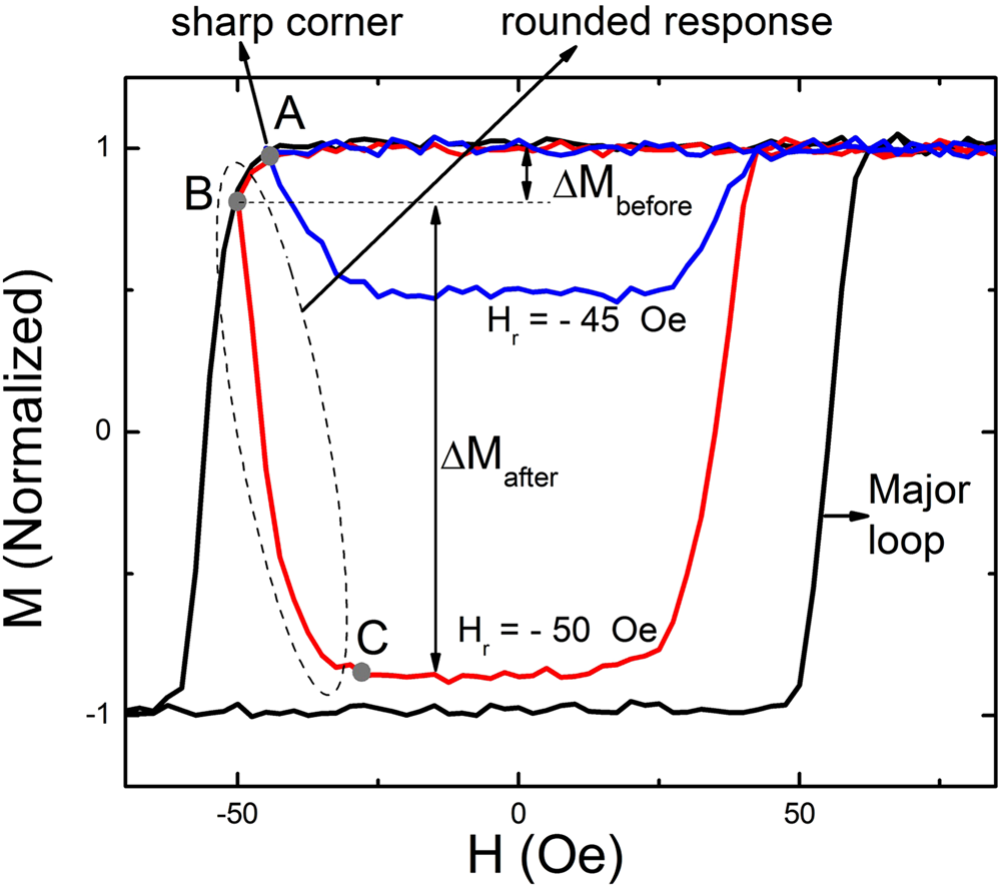
Minor loop features. Major hysteresis loop and two representative minor loops with different *H*_*r*_ (−45 and −50 Oe) but the same sweep rate of 12.5 Oe/s. A sharp corner (**A**) and a regions exhibiting rounded response (A-B-C) are noted by arrows. *ΔM*_*before*_ and *ΔM*_*after*_ are denoted for the case of *H*_*r*_ = −50 Oe.

A major loop and two representative minor loops measured with different reversing field (*H*_*r*_) at a fixed sweep rate of 12.5 Oe/s via the magnetic domain area analysis is plotted in Fig. 1. The effective sweep rate is determined by taking a field step of 2.5 Oe and a dwelling time of 0.2 s per each step into account. To measure the major loop, the applied field was ramped between +250 Oe and −250 Oe with the maximum applied field has been checked to be strong enough to saturate the film at this sweep rate without leaving any unreversed microscopic magnetic domains within the field of view. The confirmation of non-existence of unreversed magnetic domains should be carefully examined to exclude the possibility of pre-existing domain effect^6,19,20^. The minor loops were determined by ramping applied field from the positive maximum field (+250 Oe) to a negative *H*_*r*_ and then, by reversing the field to the positive maximum field. It took about 50 s to measure one minor loop at this sweep rate. All the loops were monitored for an observation area of 2.56×1.92 mm^2^. The minor loops show decreases of *M* upon increasing *H* just after reversing the field at *H*_*r*_, as denoted in the figure for the two cases of *H*_*r*_ = −45 and −50 Oe. Then the decrease of *M* slows down until *M* reaches a plateau region until coming back to a positive saturation, forming an’unusual’ rounded hysteresis response as previously observed by Cheng *et al*.^5^. The amount of magnetization reversal before and after *H*_*r*_ are defined as *ΔM*_*before*_ = *M*_*S*_ − *M(H*_*r*_) (between point A and B) and *ΔM*_*after*_ = *M(H*_*r*_*) − M*_*plateau*_ (between point B and C), as in the case of *H*_*r*_ = −50 Oe in Fig. 1. The previous work reported the rounded response involved with *ΔM*_*after*_^5^, while the present study comprehensively explores the whole region around the *H*_*r*_ by considering both *ΔM*_*after*_ and *ΔM*_*before*_. In most cases, the ratio *ΔM*_*after*_*/ΔM*_*before*_ is small so that the rounded hysteresis response becomes effectively negligible, which might be the reason that the rounded response behavior has been widely considered as ‘unusual’. In the present study, it is observed that the rounded response becomes clearly manifested as the ratio *ΔM*_*after*_*/ΔM*_*before*_ substantially changes with variation of the reversing field. For example, *ΔM*_*after*_*/ΔM*_*before*_ *~ 9*.*3* for the case of *H*_*r*_ = −50 Oe, which is significantly large compared to any reported cases ranging from ~0.3^5^ to ~3^11^ so far. Note that there exists one interesting difference for the case of *H*_*r*_ = −45 Oe in Fig. 1 that the decrease of *M* mostly proceeds when *H* increases just after *H*_*r*_, *e*.*g*., *ΔM*_*after*_*/ΔM*_*before*_ becomes extremely large, shaping a very sharp corner at the nucleation unlike any other minor loops reported till now.

### Reversing field dependence

**Figure 2:**
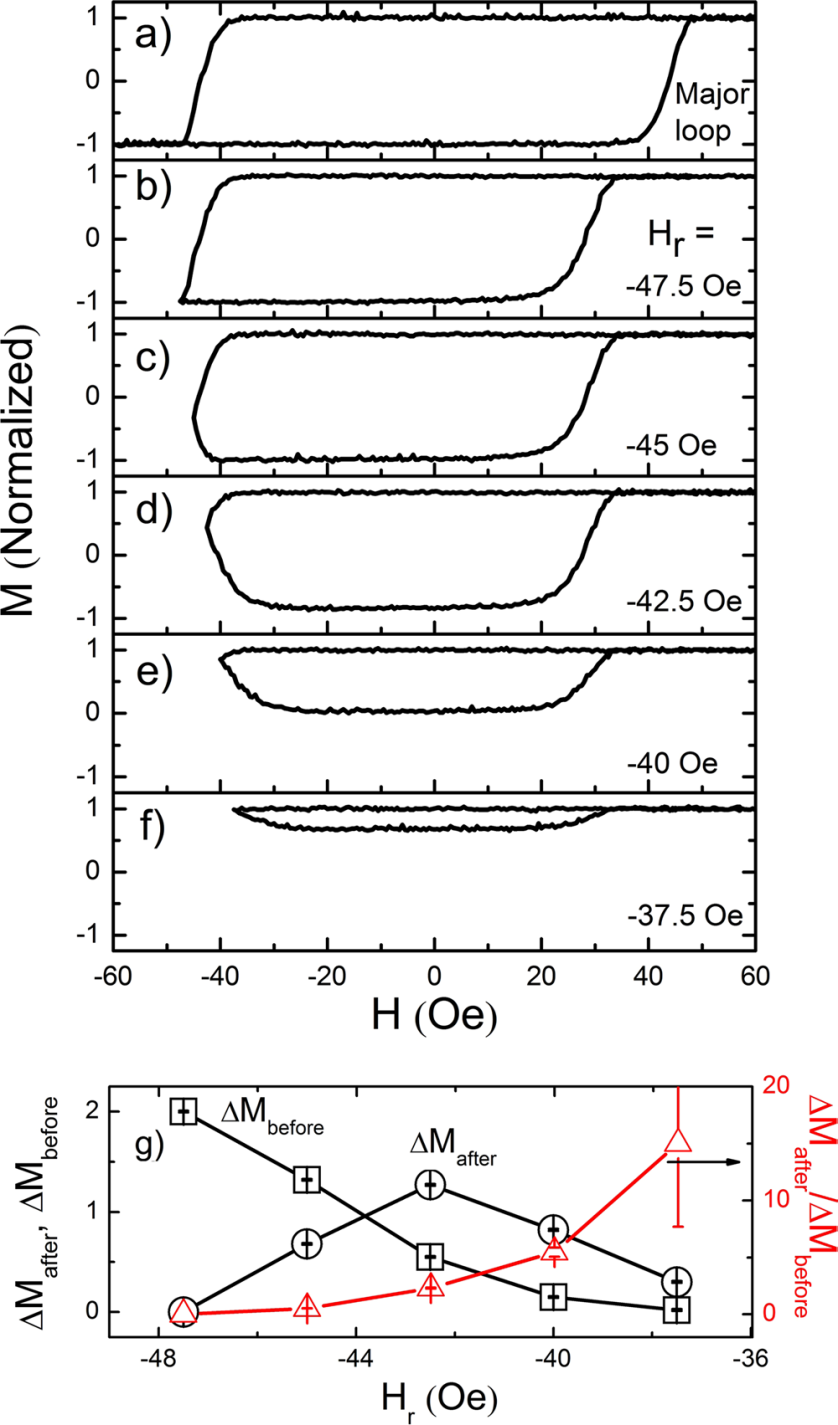
Effect of reversing field change. (**a**) A major loop and several minor loops with variation of *H*_*r*_ to be (**b**) −47.5, (**c**) −45, (**d**) −42.5, (**e**) −40, and (**f**) −37.5 Oe under the same sweep rate of 2.5 Oe/s. (**g**) *ΔM*_*after*_, *ΔM*_*before*_ and *ΔM*_*after*_*/ΔM*_*before*_ with respect to *H*_*r*_ at the sweep rate of 2.5 Oe/s.

We have investigated the rounded hysteresis response with a systematic variation of *H*_*r*_. Figure 2 shows a major loop (Fig. 2(a)) and several minor loops (Fig. 2(b–g)) with different *H*_*r*_ ranging from −47.5 to −37.5 Oe with a fixed sweep rate of 2.5 Oe/s. All the minor loops have been measured with maximum applied field of 250 Oe. It took about 40 to 1200 s to measure one minor loop depending on sweep rates. With *H*_*r*_ weaker than −47.5 Oe (Fig. 2(c–g)), *M* continues to decrease even with an increase of *H* from *H*_*r*_, exhibiting ‘unusual’ rounded hysteresis^3^. *ΔM*_*after*_ is relatively small at *H*_*r*_ = −45 Oe (Fig. 2(c)) but becomes larger for *H*_*r*_ = −42.5 Oe (Fig. 2(d)). With *H*_*r*_ = −37.5 Oe (Fig. 2(f)), the *ΔM*_*before*_ is so small that one can observe only *ΔM*_*after*_ within the measurement error, resulting in a very sharp corner around *H*_*r*_. Note that the sharp corner was also observed for a different *H*_*r*_ (−45 Oe) in Fig. 1 with a different sweep rate (12.5 Oe/s), implying the systematic study with variation of sweep rates are also required.

It is interesting to note that the ‘unusual’ rounded behavior becomes more dominant with respect to increasing *H*_*r*_ as in Fig. 2(g), where *ΔM*_*before*_, *ΔM*_*after*_, and *ΔM*_*after*_*/ΔM*_*before*_ are plotted with variation of *H*_*r*_. It is clearly observed that the ratio *ΔM*_*after*_*/ΔM*_*before*_ exhibits a monotonic increase with respect to the *H*_*r*_. The *ΔM*_*before*_ shows a monotonic decrease while *ΔM*_*after*_ is not monotonic but showing a maximum at *H*_*r*_ = −42 Oe. Thus, it is expected that the monotonic increase of the *ΔM*_*after*_*/ΔM*_*before*_ is mainly determined by the monotonic decrease of *ΔM*_*before*_.

For further details, we have plotted both *H* and *M* vs *t* as in the Supplementary Note 1, where it is observed that magnetization reversal occurs mostly before *H*_*r*_ for the case of *H*_*r*_ ≤ −7.5 Oe. It is generally observed in a ferromagnetic system that the nucleation on the decreasing branch begins before *H*_*r*_, as seen in the Supplementary Figure 1(a) for the major loop. This behavior is widely observed in other experimental results even for minor loops^5–7,11^. It should be noted that it is possible as well to have a delayed nucleation on the increasing branch of *H* > *H*_*r*_, as demonstrated in the Supplementary Figure 1(f), where it is expected to have magnetization reversal mostly occurring after *H*_*r*_, resulting in an extremely large *ΔM*_*after*_*/ΔM*_*before*_ and thus, surprisingly forming a sharp corner. We consider that the novel property of the minor hysteresis response with the sharp corner might provide an opportunity to explore further magnetic device characteristics operating along minor loops.

### Sweep-rate dependence

**Figure 3:**
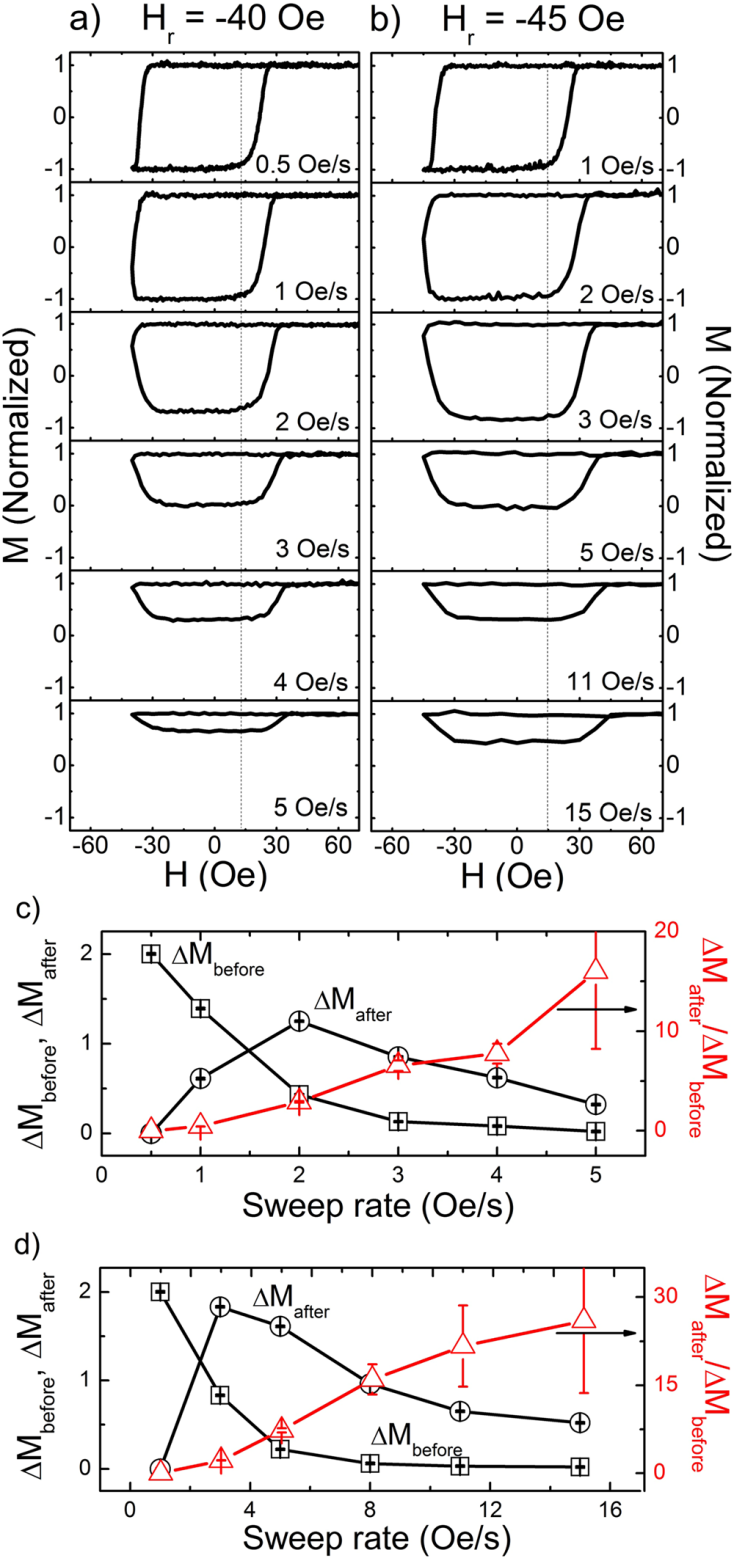
General appearance of minor loop behavior. Minor loops with variation of sweep rate (**a**) under the fixed *H*_*r*_ = −40 Oe and (**b**) −45 Oe. Sweep rate dependence of *ΔM*_*after*_ (open square), *ΔM*_*before*_ (open circle) and *ΔM*_*after*_*/ΔM*_*before*_ (solid triangle) at (**c**) *H*_*r*_ = −40 Oe and (**d**) −45 Oe.

We have investigated as well the sweep rate dependence of the rounded response of minor loops. First, sweep rates were varied from 0.5 to 5 Oe/s, while keeping the same *H*_*r*_ = −40 Oe, as in Fig. 3(a). Then, for comparison, sweep rates were also varied from 1 to 15 Oe/s with *H*_*r*_ = −45 Oe as in Fig. 3(b). In Fig. 3(a), with the sweep rate of 0.5 Oe/s, the minor loop has a shape almost similar to a major loop. If a sweep rate is greater than 1 Oe/s, it is observed that hysteresis loop area becomes smaller with a tendency for the loops to move upward. The width of the minor loops increases with respect to the sweep rate, as clearly observed in a relative comparison to the dotted line in the figure. The increase of the loop width is frequently observed as well in case of the major loop, known as the Steinmetz law^21–25^, which is originated by the delayed response of the ferromagnetic system under cycling fields. We have confirmed by direct domain observation that there exists a similar effect of delayed nucleation process even in minor loop responses, explaining the increased minor loop width.

The rounded behavior is found to become more dominant, i.e. the ratio *ΔM*_*after*_*/ΔM*_*before*_ increases with respect to the sweep rate as depicted in Fig. 3(c,d), where *ΔM*_*before*_, *ΔM*_*after*_, and *ΔM*_*after*_*/ΔM*_*before*_ are plotted with variation of the sweep rate. It is clearly observed that the ratio *ΔM*_*after*_*/ΔM*_*before*_ monotonically increases with respect to the sweep rate for both cases of *H*_*r*_ = −40 Oe (Fig. 3(c)) and −45 Oe (Fig. 3(d)), as in the case of *H*_*r*_ variation in Fig. 2(g). In case of Fig. 3(c), the *ΔM*_*before*_ shows a monotonic decrease while *ΔM*_*after*_ is not monotonic but showing a maximum at 2 Oe/s, which is again explainable based on the fractional reversal in case of faster sweep rates. Similar trend is found as well in Fig. 3(d).

In case of *H*_*r*_ = −40 Oe, for sweep rates greater than 4 Oe/s, the nucleation start after *H*_*r*_ so that the sharp corner at nucleation is clearly observed again. The same trend is confirmed for the case of *H*_*r*_ = −45 Oe, where a faster sweep rate is required to have a similar trend of minor loop behavior as in Fig. 3(b). The observed hysteresis responses are clearly related to the delayed response of the system, which can be explained based on the domain observation results in the next part.

We have further investigated time-dependent H and M at various sweep rates (0.5–5 Oe/s) as demonstrated in the Supplementary Note 2, where a response with relatively different delays via the microscopic domain dynamics under slow or fast sweep rate is found to exhibit the rounded minor loop response.

### Domain observations

**Figure 4:**
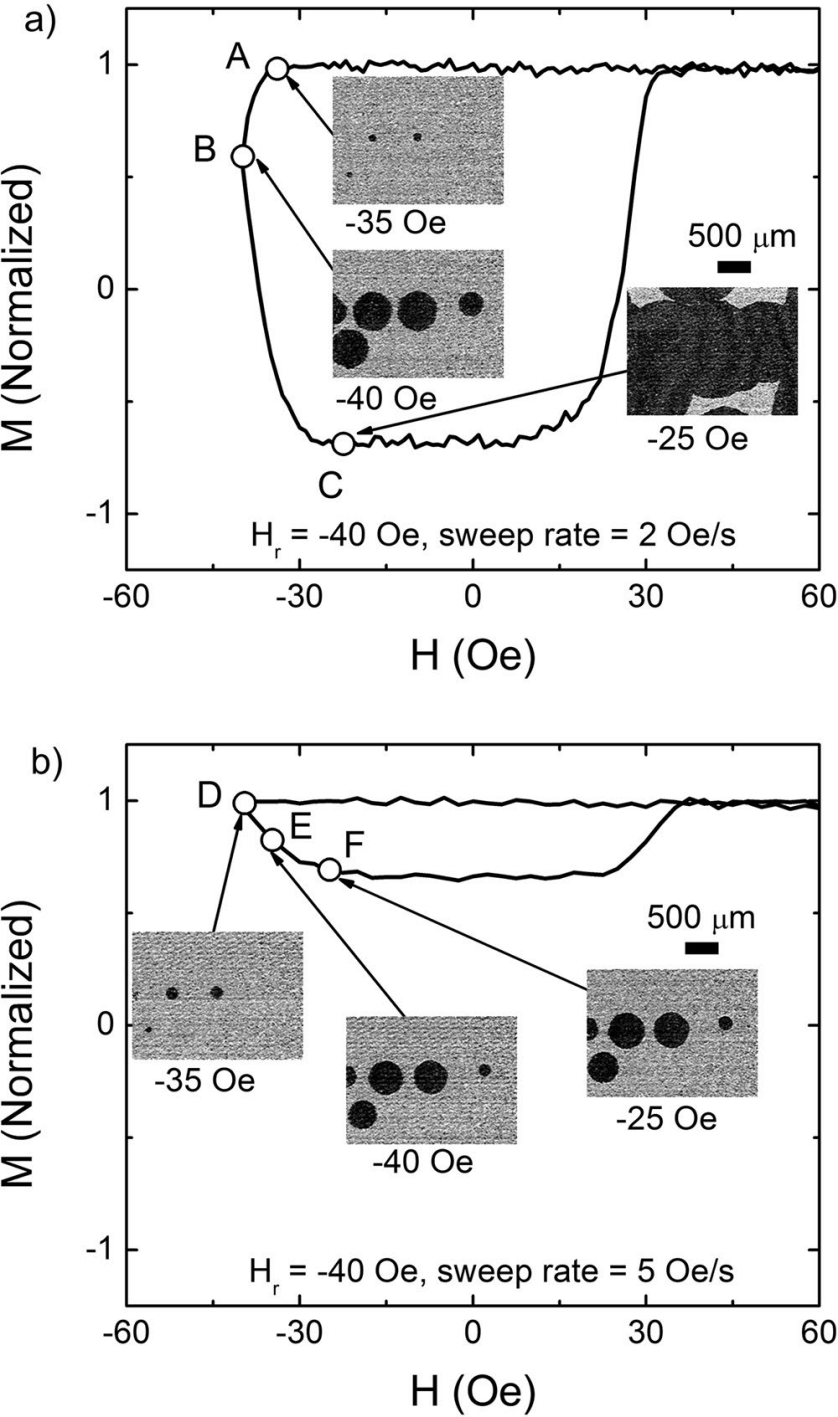
Minor loop and magnetic domains. Minor loops with *H*_*r*_ = −40 Oe for different sweep rate of (**a**) 2 Oe/s and (**b**) 5 Oe/s. Corresponding magnetic domains for different points along the minor loops from A-F are illustrated. Scale bars are in the figure.

**Figure 5:**
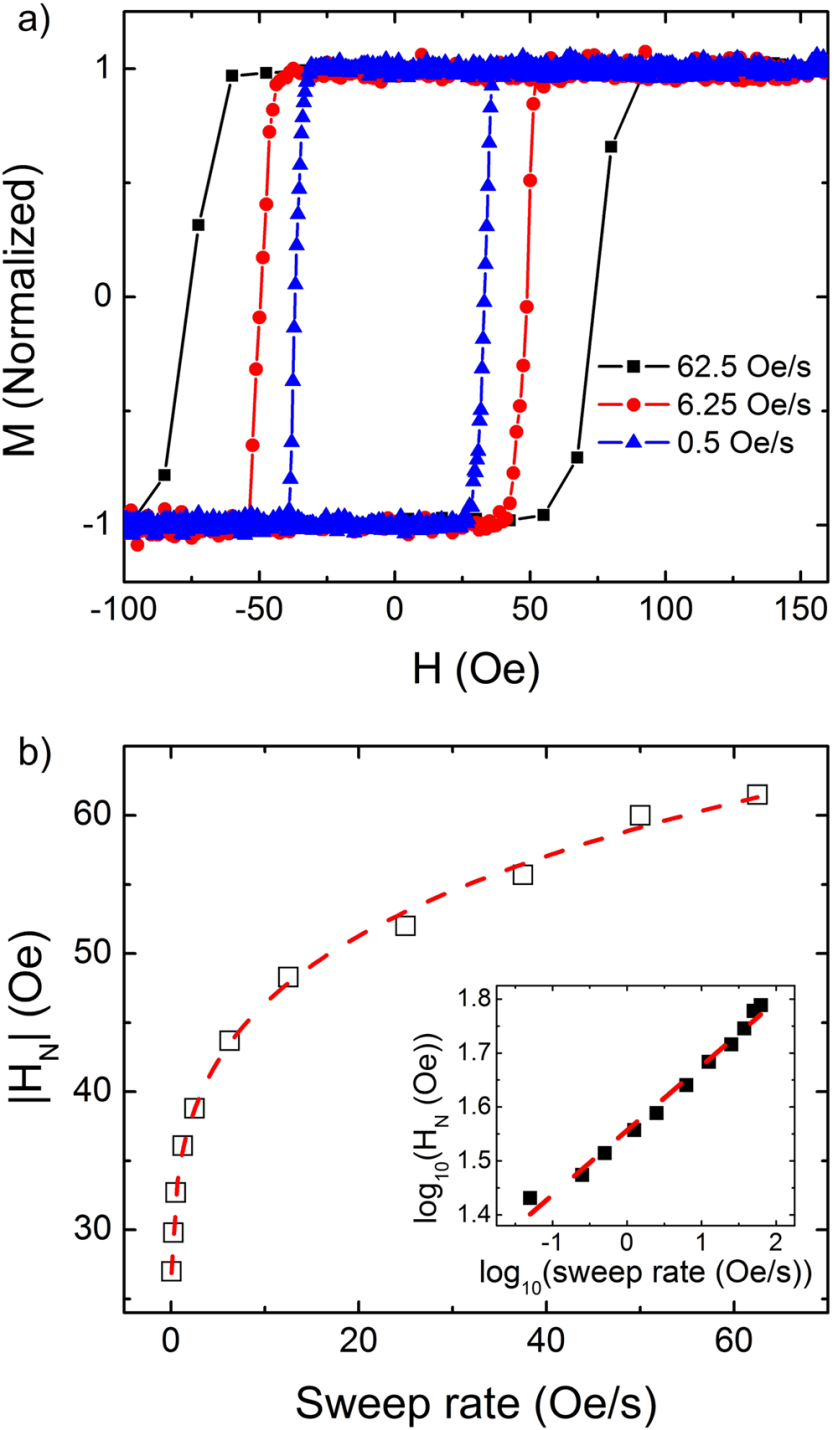
Sweep-rate dependent major loop and nucleation. (**a**) Major loops measured at various sweep rates from 0.5 to 62.5 Oe/s. (**b**) Nucleation field as a function of sweep rate (open square). Dashed line is a fitting by Eq. (1). The inset is the same graph on a log-log scale.

To understand the observed rounded response and sharp corners of minor hysteresis loops, we have systematically analyzed microscopic domain structures during the magnetization reversal. Domain structures together with two minor loops for the same observation area are illustrated in Fig. 4, for different sweep rates of 2 Oe/s (Fig. 4(a)) and 5 Oe/s (Fig. 4(b)) with the same *H*_*r*_ = −40 Oe. The two cases are selected to represent the two unusual behaviors of the rounded response (Fig. 4(a)) and the sharp corner at the nucleation (Fig. 4(b)).

In Fig. 4(a), domain patterns at the nucleation corner (A), at the reversing field (B), and at the plateau (C) are presented. At the nucleation (A), it is observed that three tiny black domains are nucleated at −35 Oe, which is followed by two more nucleations and subsequent domain wall expansion, leading to a decrease of *M* from A to B. After *H*_*r*_, the domains continue to expand under increasing *H* to −25 Oe (point C), resulting in a decrease of *M* as in the corresponding minor loop in the figure. In a similar way, all rounded responses observed in the present study are explained by the continued wall expansion and more nucleation.

In Fig. 4(b), when *H* decreases to *H*_*r*_ = −40 Oe (D), one can see several domains nucleated in the observation area, where the nucleation field (*H*_*N*_) is easily defined to be equal to *H*_*r*_. After reaching *H*_*r*_, *H* increases toward *H*_*max*_, however, the nucleated domains continue to expand (E) until *H* reaches a plateau region at *H* = −25 Oe (F), leading to the decrease of *M* as the same as the *ΔM*_*after*_ upon increase of *H*, finally forming a sharp corner of the corresponding minor loop.

From the observation, we have confirmed that the domain expansions and more nucleations are continued even under increasing *H* from *H*_*r*_, effectively generating a delayed response, which is measured to be a rounded minor loop response. It is also observed that the *H*_*N*_ sensitively depends on the field sweep rate and even could be tuned to be equal to the *H*_*r*_, at which a sharp nucleation corner is shaped in the loop.

We have also measured the time-dependent magnetization reversal of the sample under different applying fields, of which results are presented in the Supplementary Note. 3. We have discovered that for the relaxation behavior under different fields falls into a universal curve, with the reversal time is normalized by the half-reversal time. The S-shape of the relaxation curve implies that the reversal is dominantly mediated by domain wall propagation rather than domain nucleation.

### Nucleation field

We have also examined the *H*_*N*_ behavior depending on the sweep rate. In Fig. 5(a), three major loops under different sweep rates varying from 0.5 Oe/s to 62.5 Oe/s are plotted with the maximum applied field of 250 Oe. With increasing sweep rates one can clearly see the increase of the $|{H}_{N}|$ as well as the coercivity and loop area, as expected from the Steinmetz law^21–25^. It should be mentioned that *H*_*N*_ measured for the major loop at a certain sweep rate is expected to be the same as *H*_*N*_ of minor loops at the same sweep rate, since all the minor loops were measured by the same positive maximum field (+250 Oe).

The true microscopic *H*_*N*_ at which domains start to nucleate is not easy to experimentally determine due to the limited spatial resolution. In the present work, for simplicity, *H*_*N*_ is defined as the value of *H* when *M(H)* is reduced to be 90% of the saturated state.

*H*_*N*_ values with respect to the sweep rate are plotted in Fig. 5(b), which is fitted by

1 $$|{H}_{N}|=|{H}_{N0}|+{K}_{N}{(\frac{dH}{dt})}^{\eta },$$

where *H*_*N0*_, *K*_*N*_ and *η* are the static nucleation field, the scaling factor for nucleation, and the scaling exponent for the nucleation, respectively^21,25^ The best fitting, which is also demonstrated in the inset figure, provides |*H*_*N0*_| = 19.2 ± 1.8 Oe, *K*_*N*_ = 15.6 ± 1.9, and *η* = 0.24 ± 0.02. *K*_*N*_ and *η* represent the overall nucleation behavior involved with microscopic domain configuration during the field cycling. Compared to the case of Co/Pt multilayer (*K*_*N*_ ~ 35 and *η* = 0.5), CoFeB/Pd multilayer in the present study exhibits a relatively lower value of *K*_*N*_, implying a more simplified (or complex) domain configuration. The different *η* value also implies the nucleation process here might fall into a different universality. Further discussion on *K*_*N*_ and *η* is not a major interest in this work. Rather, we focus on the static nucleation field *H*_*N0*_, since *H*_*N0*_ is supposed to be irrespective of the sweep rate, which could be used as a useful parameter in explaining the observed minor loop behavior.

### Domain wall velocity

We have analyzed the domain wall (DW) velocity as well. To measure the DW velocity under different magnetic fields, we adopted a similar technique as in ref.^26–29^. The film was first positively saturated then a small negative field about *H*_*C*_ was applied to generate a small domain in the field of view. Then, an external negative field is applied to drive the DW to expand. The domain images were recorded and subtracted from one another to determine the distance swept by the DW at each time interval (0.2 s), from which the DW velocity was calculated. The applied field at which DW velocity was measured is varied from −50 Oe to −10 Oe, covering a range of *H*_*N*_ and *H*_*r*_ in all cases discussed above. The determined DW velocity is presented in the Supplementary Figure 3(a). It is observed that the DW still slowly moves at *H* = −10 Oe. Thus, *H*_*depin*_ is estimated to be weaker than −10 Oe. It should be noted that the depinning field is much weaker than the static nucleation field (*H*_*N0*_ = −19.2 ± 1.8 Oe). Therefore, it is expected to have a continued DW motion even in the case when *H* increases from *H*_*r*_, eventually leading to the rounded response of the minor hysteresis loop, once *H* is still stronger than *H*_*depin*_. Moreover, the reversed domains might continue to expand when the *H* direction is still parallel to the *M* of the reversed domains, still providing an energetic pressure by the Zeeman energy. From this, we conclude that the ‘unusual’ rounded minor loop response could be a general aspect of the minor loop response, appearing when the ferromagnetic system has a weaker *H*_*depin*_ than *H*_*N0*_. It should be also mentioned that the DW dynamics in the observation condition is well described by the creep phenomenon. The creep exponent of −1/4^26–29^ is confirmed in the Supplementary Figure 3(b).

## Discussions

Now we can build a physical picture and comprehensive understanding of the observed unusual minor hysteresis loop behavior such as rounded response and sharp corner. In case of the fixed sweep rate but different *H*_*r*_ as in Fig. 2, the *H*_*N*_ is found to be the same (~−37.5 Oe) for all cases. When *H*_*r*_ is small enough, (<−47.5 Oe), there seems to be enough time for the reversed domains to expand covering the whole observation area before *H* reaches *H*_*r*_ and thus, *ΔM*_*after*_ cannot be observed. For a larger *H*_*r*_, for example, as in *H*_*r*_ = −45 Oe, *H* is reversed before the domain completes to cover the whole observation area. After *H*_*r*_, the reversed domains continue to expand by the creep motion due to thermally assisted activation under external fields stronger than *H*_*depin*_. The continuing DW expansion allows us to observe *ΔM*_*after*_. With increase of *H*_*r*_, the reversed domains are provided with a relatively shorter time to expand before *H*_*r*_, implying a smaller *ΔM*_*before*_ as seen indeed in Fig. 2(c–e). If *H*_*r*_ becomes close to *H*_*N*_ ~ −37.5 Oe, *ΔM*_*before*_ cannot be observed but only *ΔM*_*after*_ is observable, resultantly shaping a sharp corner at nucleation (Fig. 2(f)).

In case of sweep rate variations with fixing *H*_*r*_, cycling with a faster sweep rate produces a minor loop with a higher |*H*_*N*_|. If sweep rate is slow enough, e.g., 0.5 Oe/s as in Fig. 3, |*H*_*N*_| becomes lowered (~29 Oe) so that there exists enough time for the nucleated domains to expand to cover the whole observation area before ramping *H* back. If a sweep rate is fast enough so that *H*_*N*_ becomes stronger and eventually comparable to *H*_*r*_, as in the case of Fig. 3 (5 Oe/s sweep rate), the domain expansion before *H*_*r*_ becomes negligible with squeezing an observable *ΔM*_*before*_. In this case, domains expand mostly after *H*_*r*_ while *H* is stronger than *H*_*depin*_, generating *ΔM*_*after*_ and forming a minor loop with a sharp corner at nucleation. It should be mentioned that such minor loop responses are expected to be generally observed when *H*_*N*_ is comparable to *H*_*r*_ and stronger than *H*_*depin*_.

At a fixed sweeping rate, if the magnetic field is reversed after domain nucleation but the magnetization has not yet significantly decayed, the decay of the magnetization mostly occurs along the increase of the field, forming a ‘sharp’ corner at the reversing point. If the magnetization evolution does not stop but continues to decay slowly along the increase of the field, a ‘rounded’ corner is formed at the reversing point.

Compared to previous reports on the unusual rounded response behavior, the observed roundedness in this work is significantly large, which is possible due to the relatively weaker *H*_*depin*_ compared to *H*_*N*_, *H*_*N0*_, and *H*_*r*_. For the case of *H*_*N0*_ much stronger than *H*_*depin*_, both the rounded response and the sharp corner behaviors are believed to be observable at a relatively slow sweep rate. For *H*_*depin*_ is approximate to *H*_*N0*_, the rounded response is expected to be only observed with relatively fast sweep rates.

We have performed the same experimental test for (Co/Pt)_5_ multilayer with a perpendicular magnetic anisotropy as well to show that the rounded minor hysteresis is associated indeed with the field seep rate, which might be applied in general regardless of the material selection. The details are given in the Supplementary Notes 5 and 6. In summary, we have systematically investigated the minor loop response behavior by quantitative analysis of field- and time-dependent magnetization reversal in CoFeB/Pd multilayer with a perpendicular magnetic anisotropy. The ‘unusual’ minor hysteresis responses such as the rounded response and the sharp corner have been intensively examined via a direct magnetic domain observation, which is found to be clearly explainable based on domain wall dynamics with a careful consideration of *H*_*N*_, *H*_*N0*_, *H*_*r*_, and *H*_*depin*_. We believe that the minor loop response patterns with rounded/sharpened reversing behavior could be a general feature of minor loop patterns, providing an important tool in detailed analysis and design of the ferromagnetic systems under minor cycles.

## Methods

The ferromagnetic thin film of (4-Å CoFeB/10-Å Pd)_4_ multilayer with a perpendicular magnetic anisotropy was fabricated by a DC magnetron sputtering. Details of sample fabrication condition can be found elsewhere^30^. Magnetic domains were imaged along the minor hysteresis loop by the magneto-optical Kerr microscopy^31,32^. The schematic diagram and description of the microscopy is in the Supplementary Note 7. Direct observation of the domain pattern evolution along minor hysteresis loops was carried out under an external field perpendicular to the film plane. The domain images were analyzed simultaneously to produce Kerr intensity with respect to the time *(t)* as well as with respect to the field *(H)*. Then, Kerr intensities were normalized by the saturated Kerr intensity to achieve normalized magnetization *M(t)* and *M(H)* with respect to the time and the field, respectively.

### Data availability

The data that support the findings of this study are available from the corresponding authors upon reasonable request.

## Electronic supplementary material

Supplementary Information

## References

[1] Carey R, Isaac ED, Thomas BWJ (1966). Simultaneous magneto-optic recording of hysteresis loops from both surfaces of a magnetic film. Nature.

[2] G Bertotti, Hysteresis in Magnetism: For Physicists, Materials Scientists, and Engineers (Academic Press, San Diego, 1998).

[3] Liu XD, Berger A, Wuttig M (2001). Stability of the perpendicular magnetic anisotropy of ultrathin Ni films on Cu(100) upon multiple magnetization reversals. Phys. Rev. B.

[4] Nolting F (2000). Direct observation of the alignment of ferromagnetic spins by antiferromagnetic spins. Nature.

[5] Cheng XM, Nikitenko VI, Shapiro A, Shull JRD, Chien CL (2006). Unusual magnetization reversalin (Co/Pt)_4_ multilayers with perpendicular anisotropy. J. Appl. Phys..

[6] Davies JE (2004). Magnetization reversal of Co/Pt multilayers: Microscopic origin of high-field magnetic irreversibility. Phys. Rev. B.

[7] Berger A, Mangin S, McCord J, Hellwig O, Fullerton EE (2010). Cumulative minor loop growth in Co/Pt and Co/Pd multilayers. Phys. Rev. B.

[8] Windsor YW, Gerber A, Karpovski M (2012). Dynamics of successive minor hysteresis loops. Phys. Rev. B.

[9] Meilikhov EZ, Farzetdinova RM (2012). Creeping of minor hysteresis loops in Co thin films. J. Appl. Phys..

[10] Meilikhov EZ, Farzetdinova RM (2013). Cumulative growth of minor hysteresis loops in the Kolmogorov model. J. Exp. Theor. Phys..

[11] Robb DT (2008). Evidence for a dynamic phase transition in [Co/Pt]_3_ magnetic multilayers. Phys. Rev. B.

[12] Dennis CL (2015). Internal magnetic structure of nanoparticles dominates time-dependent relaxation processes in a magnetic field. Adv. Funct. Mat..

[13] Pike CR, Roberts A, Verosub KL (1999). Characterizing interactions in fine magnetic particle systems using first order reversal curves. J. Appl. Phys..

[14] Pike CR (2003). First-order reversal-curve diagrams and reversible magnetization. Phys. Rev. B.

[15] Stancu A, Pike C, Stoleriu L, Postolache P, Cimpoesu D (2003). Micromagnetic and Preisach analysis of the First Order Reversal Curves (FORC) diagram. J. Appl. Phys..

[16] John AB, Frederick M (1973). Barkhausen noise power versus size of a minor hysteresis loop. J. Appl. Phys..

[17] Kobayashi S, Takahashi S, Shishido T, Kamada Y, Kikuchi H (2010). Low-field magnetic characterization of ferromagnets using a minor-loop scaling law. J. Appl. Phys..

[18] O’Grady K, Greaves SJ (1994). Minor hysteresis loop effects in magnetic materials. J. Magn. Magn. Mater..

[19] Windsor YW, Gerber A, Korenblit I, Ya., Karpovski M (2013). Time dependence of magnetization reversal when beginning with pre-existing nucleation sites. J. Appl. Phys..

[20] Choe S-B, Shin S-C (2001). Complete magnetizing field relating with magnetization reversal dynamics. J. Magn. Magn. Mater..

[21] Colaiori F, Durin G, Zapperi S (2006). Loss Separation for dynamic hysteresis in ferromagnetic thin films. Phys. Rev. Lett..

[22] Steinke N-J, Moore TA, Mansell R, Bland JAC, Barnes CHW (2012). Nonuniversal dynamic magnetization reversal in the Barkhausen-dominated and mesofrequency regimes. Phys. Rev. B.

[23] Handoko D, Lee S-H, Lee KM, Jeong J-R, Kim D-H (2013). Comparison of hysteresis loop area scaling behavior of Co/Pt multilayers: Discrete and continuous field sweeping. J. Magn. Magn. Mater..

[24] Raquet B, Mamy R, Ousset JC (1996). Magnetization reversal dynamics in ultrathin magnetic layers. Phys. Rev. B.

[25] Handoko D (2016). Dynamic scaling behavior of nucleation and saturation field during magnetization reversal of Co/Pt multilayers. IEEE Trans. Magn..

[26] Choi YH (2014). Field-induced domain wall motion of amorphous [CoSiB/Pt]_N_ multilayers with perpendicular anisotropy. J. Appl. Phys..

[27] Iunin YL (2007). Asymmetric domain nucleationand unusual magnetization reversalin ultrathin Co films with perpendicular anisotropy. Phys. Rev. Lett..

[28] Lemerle S (1998). Domain wall creep in an Ising ultrathin magnetic film. Phys. Rev. Lett..

[29] Kim JY, Kim K-J, Choe S-B (2009). Temperature dependence of domain-wall creep in Pt/CoFe/Pt films. IEEE. Trans. Magn..

[30] Ngo D-T (2014). Perpendicular magnetic anisotropy and the magnetization process in CoFeB/Pd multilayer films. J. Phys. D: Appl. Phys..

[31] Kim D-H, Choe S-B, Shin S-C (2003). Direct observation of Barkhausen avalanche in Co thin films. Phys. Rev. Lett..

[32] Quach D-T, Kim D-H (2014). Direct magnetic domain observation of magnetization reversal with pre-existing nucleation sites in Co/Pt multilayer. IEEE Trans. Magn..

