# Supplementary material for: Minor hysteresis patterns with a rounded/sharpened reversing behavior in ferromagnetic multilayer
Source: Sci Rep. 2018 Mar 13;8:4461. doi: 10.1038/s41598-018-22810-y (PMC5849688; doi:10.1038/s41598-018-22810-y)
Supplement: Supplementary file 1 — Supplementary Information [file 41598_2018_22810_MOESM1_ESM.pdf]

Supplementary information for

Minor hysteresis patterns with a rounded/sharpened reversing behavior in ferromagnetic multilayer

Duy-Truong Quach,[1,2,3*] Duc-Thang Pham,[2] Duc-The Ngo,[4] The-Long Phan,[5] Seung-Young Park,[6] Sang-Hyuk Lee[3], and Dong-Hyun Kim[3†]

[1]*Faculty of Basic Sciences, University of Transport and Communications, Hanoi 10000, Vietnam*

[2]*Faculty of Engineering Physics and Nanotechnology, VNU University of Engineering and Technology, Hanoi 10000, Vietnam*

[3]*Department of Physics, Chungbuk National University, Cheongju 28644, South Korea*

[4]*School of Materials, University of Manchester, Manchester M13 9PL, United Kingdom*

[5]*Department of Physics and Oxide Research Center, Hankuk University of Foreign Studies, Yongin 17035, South Korea*

[6] *Spin Engineering Physics Team, Korea Basic Science Institute, Daejeon 34133, South Korea*

Supplementary Note 1. Reversing-field variation

H and *M* vs *t* is plotted in the Supplementary Figure 1, corresponding to each value of H_r in Fig. 2. The time is set to be zero when *H* reaches H_r. Magnetization reversal occurs mostly before H_r for the case of $H_r \leq$ -47.5 Oe, while the reversal occurs mostly after H_r for the case of $H_r \geq$ -40 Oe, leading to an overall increase of the ratio $\Delta M_{after}/\Delta M_{before}$ with respect to the H_r. The maximal ΔM_{after} observed in Fig. 2(g) is due to the external field around H_r is not enough to trigger a massive magnetization reversal, only reversing a fractional region as in the case of the Supplementary Figure 1(e) and (f).

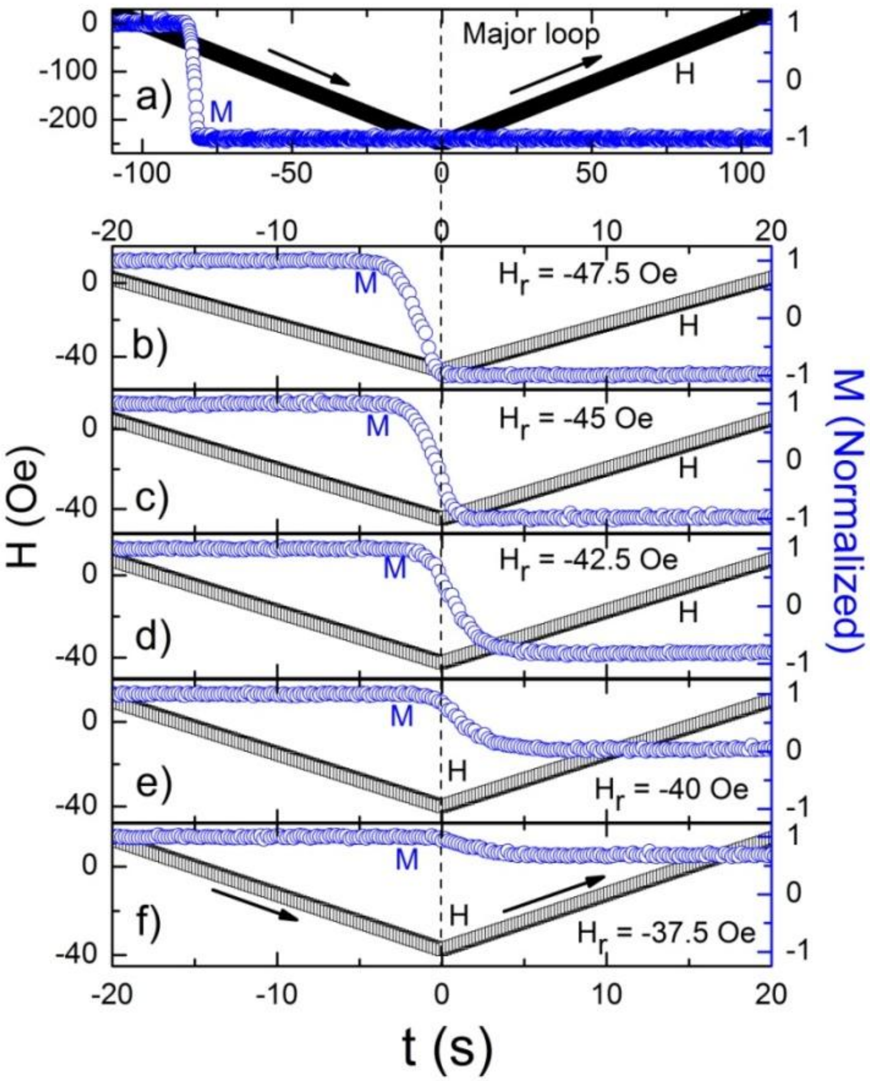

Supplementary Figure 1. Reversing-field-dependent magnetization dynamics. (a) Magnetization (blue open circle) and external field profile (black solid) with respect to the time for the case of major loop. Magnetization (blue open circle) and external field profiles (black open square) with respect to the time for different H_r of (b) -47.5, (c) -45, (d) -42.5, (e) -40, and (f) -37.5 Oe. The arrows denote decrease (before t = 0) and increase (after t = 0) of H. The vertical dashed line is a guide separating the graph into two parts as before (left) and after H_r (right).

Supplementary Note 2. Sweep-rate variation

Time-dependent H and M at various sweep rates are plotted in the Supplementary Figure 2 for the case of H_r = -40 Oe, corresponding to the case of Fig. 4(a). At a slow sweep rate of 0.5 Oe/s, the magnetization reversal occurs before H_r, forming a hysteresis loop shape similar to a major loop. At faster sweep rates, magnetization reversal occurs partly after H_r due to delayed response by the microscopic domain dynamics, providing the rounded minor loop response. At a fast sweep rate of 5 Oe/s, the magnetization reversal occurs completely after H_r, shaping a very sharp corner at nucleation as seen in Fig. 3(a).

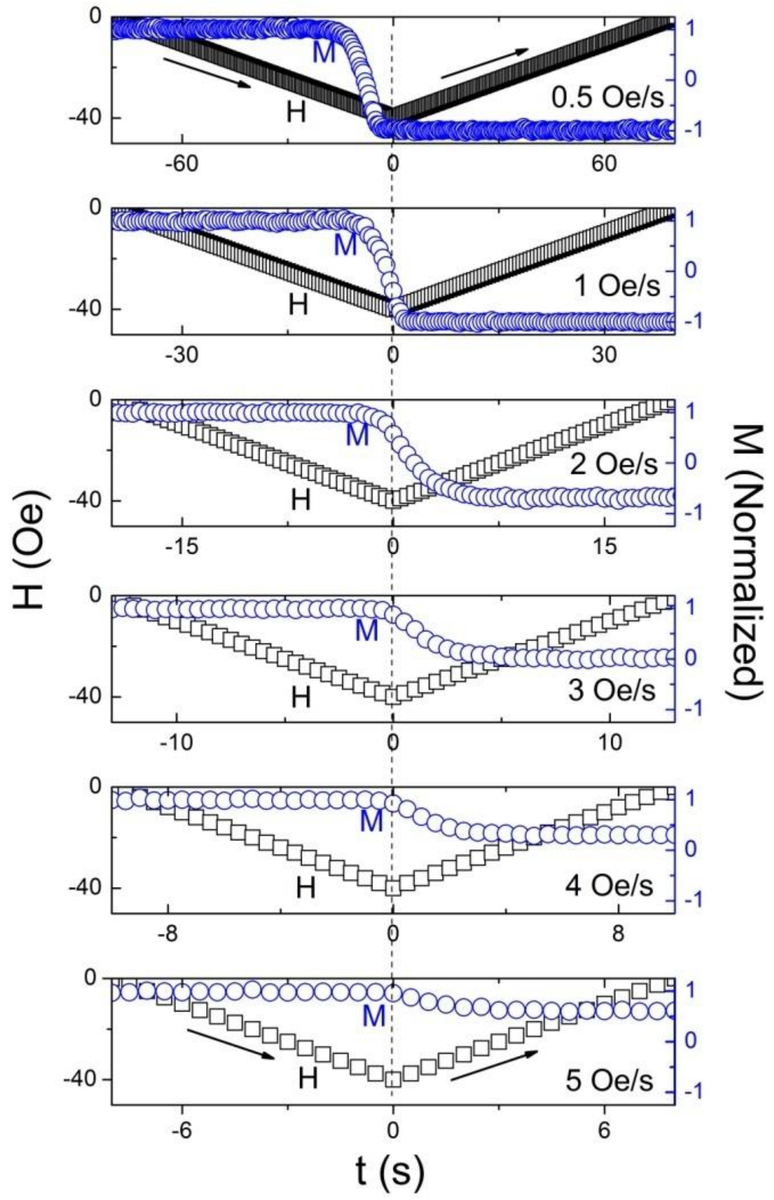

Supplementary Figure 2. Sweep-rate-dependent magnetization dynamics. Magnetization (blue open circle) and external field profiles (black open square) with respect to the time under different sweep rates ranging from 0.5 to 5 Oe/s. The arrows denote decrease (before t = 0) and increase (after t = 0) of H. The vertical dashed line is a guide separating the graph into two parts as before (left) and after H_r (right).

Supplementary Note 3. Decaying behavior

The time-dependent magnetization relaxation curves under different applied fields from -16 Oe to -36 Oe are measured, as shown in Supplementary Figure 3(a). Although the decaying behavior seems to be quite different among each others, the normalized curves with time t normalized by half-reversal time ($t_{1/2}$) fall into a universal one, as seen in Supplementary Figure 3(b), clearly exhibiting the S-shape decaying curve, rather than L-shape curve. The S-shape decaying curve indicates that the magnetization reversal in the present study is dominantly governed by domain wall motion rather than domain nucleation.

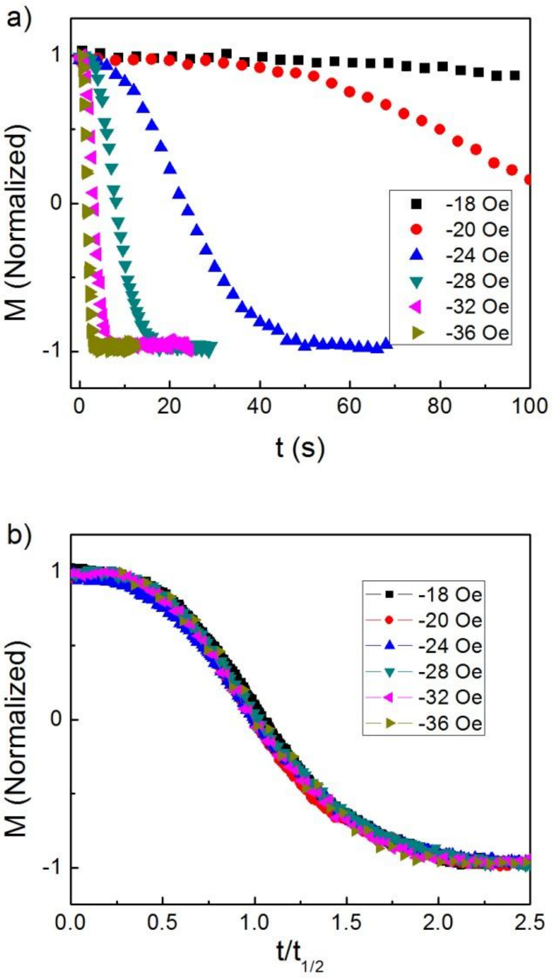

Supplementary Figure 3. (a) Time-dependent magnetization relaxation curves under different fields from -16 Oe to -36 Oe. (b) Normalized relaxation curve with the time t normalized by half-reversal time $t_{1/2}$.

Supplementary Note 4. Domain wall velocity

The DW velocity is plotted in the Supplementary Figure 4(a). The inset figure is a zoomed-in graph for the relatively weaker field region, clearly indicating the existence of the DW depinning field (H_{depin}). We have also confirmed that the DW dynamics is well described by the creep phenomenon. DW velocity is fitted, as plotted in the Supplementary Figure 4(b), by

$$v = v_0 \exp\left[-\left(\frac{U_C}{K_B T}\right)\left(\frac{H_{crit}}{|H|}\right)^{\mu}\right],$$

where v_0 is a numerical prefactor with the unit of speed, U_c is a constant related to the pinning potential strength, H_{crit} is the critical field, and μ is the creep exponent.[1-3]

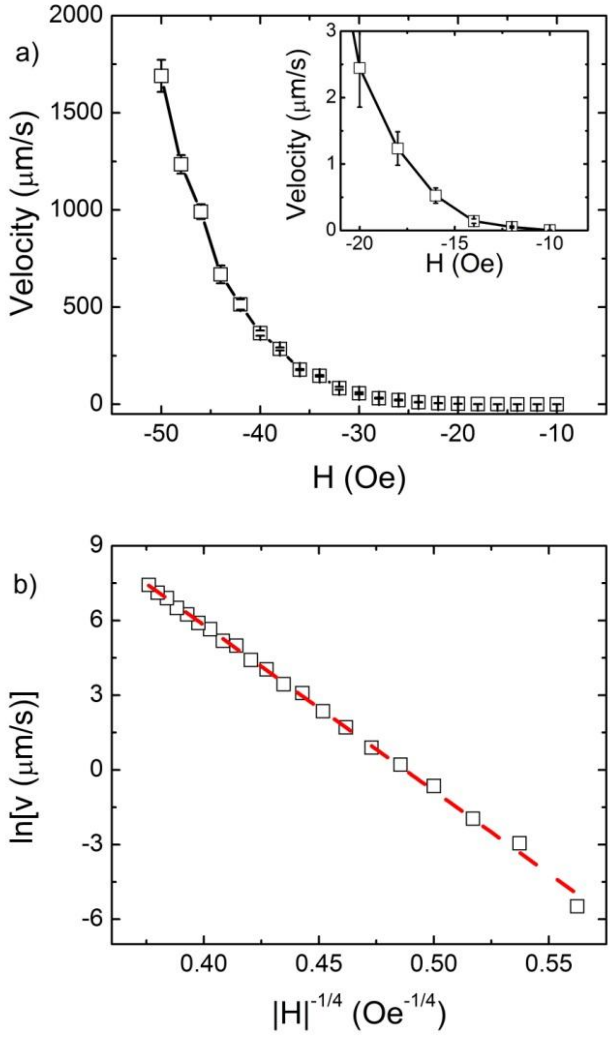

Supplementary Figure 4. Domain wall creep behavior. (a) Field-dependent DW velocity. The inset is a zoomed-in graph for the weak field region. (b) *ln*(*v*) versus $|H|^{-1/4}$ with a linear fit by the Eq. (2) (dashed line).

Supplementary Note 5. Minor loops of $(CoFeB/Pd)_4$ with variation of sweep rates

Minor loops of $(CoFeB/Pd)_4$ multilayer with variation of sweep rate from 0.125 Oe/s to 1 Oe/s with H_r = -35 Oe. The trend of the change of minor loops with variation of sweeping rate is found to be similar as in the case of H_r = -40 and -45 Oe.

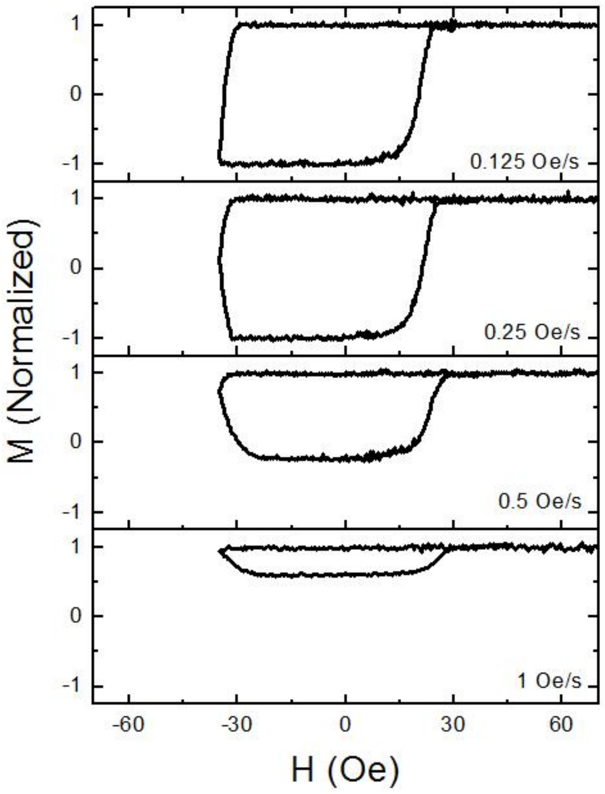

Supplementary Figure 5. Minor loops of $(CoFeB/Pd)_4$ multilayer with variation of sweep rates with H_r = -35 Oe.

Supplementary Note 6. Minor loops of $(Co/Pt)_5$

Minor loops of $(Co/Pt)_5$ multilayer at a sweep rate of 7 Oe/s with variation of H_r = -290 Oe to -330 Oe. The sharpened corner is obseved at H_r = -290 Oe, as in the case of $(CoFeB/Pd)_4$ multilayer. The result implies that the investagation method could be applied for any chosen materials without loss of generality.

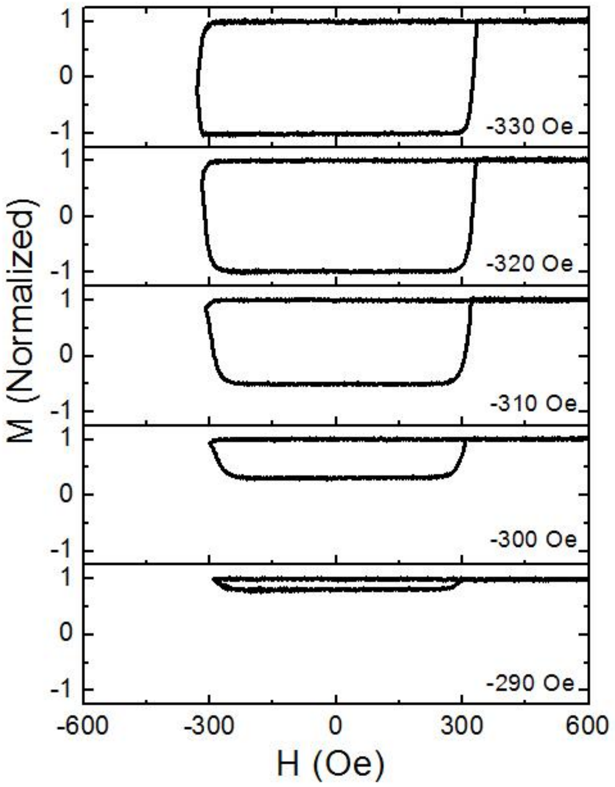

Supplementary Figure 6. Minor loops of $(Co/Pt)_5$ multilayer at a sweep rate of 7 Oe/s with variation of H_r = -290 Oe to -330 Oe.

Supplementary Note 7. Magneto-optical Kerr microscopy magnetometer

Schematic diagram of magneto-optical Kerr microscopy and measurement method are illustrated in the Supplementary Figure 7. CCD images are taken with 30 Hz under low light intensity condition, which are quantitatively processed in real-time to produce magnetic domain patterns, Kerr hysteresis loop (major/minor), and magnetic relaxation curve. The spatial resolution is 400 nm at ×1000 magnification and the temporal resolution is 33 ms. Green laser diode is used as a light source to maximize the Kerr rotation in ferromagnetic films including Co. Electromagnet is attached below the sample to exert a perpendicular magnetic field with arbitrary field profile.

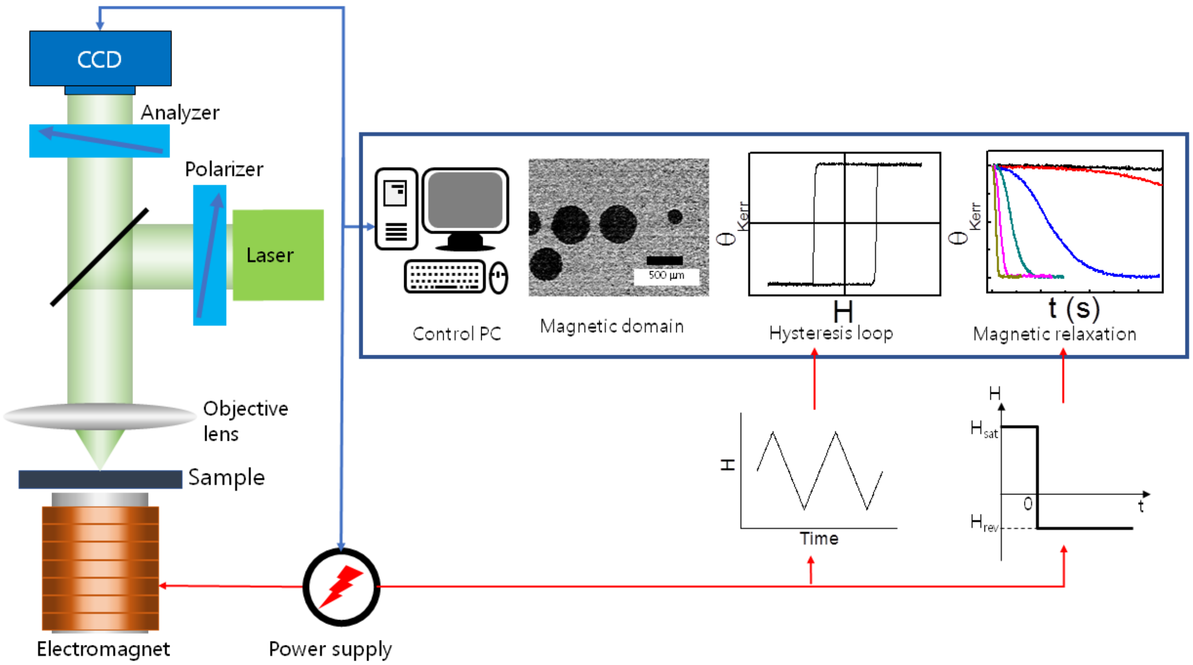

Supplementary Figure 7. Schematic diagram of magneto-optical Kerr microscopy. From quantitative analysis of CCD images, magnetic domain patterns, Kerr hysteresis loop, and magnetic relaxation behavior are measured.

References

1. Choi, Y. H., Lee, K. J., Yoon, J. B., Cho, J. H., You, C.-Y., Kim, T. W., and Jung, M. H. Field-induced domain wall motion of amorphous $[CoSiB/Pt]_N$ multilayers with perpendicular anisotropy. *J. Appl. Phys.* **115**, 183901 (2014).
2. Lemerle, S., Ferre, J., Chappert, C., Mathet, V., Giamarchi, T., and Le Doussal, P. Domain wall creep in an Ising ultrathin magnetic film. *Phys. Rev. Lett.* **80**, 849-852 (1998).
3. Kim, J. Y., Kim, K.-J., and Choe, S. -B. Temperature dependence of domain-wall creep in Pt/CoFe/Pt films. *IEEE. Trans. Magn.* **45**, 3909-3901 (2009).
